# Industrial perspectives for personalized microneedles

**DOI:** 10.3762/bjnano.14.70

**Published:** 2023-08-15

**Authors:** Remmi Danae Baker-Sediako, Benjamin Richter, Matthias Blaicher, Michael Thiel, Martin Hermatschweiler

**Affiliations:** 1 Nanoscribe Gmbh & Co, Hermann-von-Helmholtz-Platz 6, 76344 Eggenstein-Leopoldshafen, Germany

**Keywords:** 3D printing, microfabrication, microneedles, personalized medicine, transdermal drug delivery, two-photon polymerization

## Abstract

Microneedles and, subsequently, microneedle arrays are emerging miniaturized medical devices for painless transdermal drug delivery. New and improved additive manufacturing methods enable novel microneedle designs to be realized for preclinical and clinical trial assessments. However, current literature reviews suggest that industrial manufacturers and researchers have focused their efforts on one-size-fits-all designs for transdermal drug delivery, regardless of patient demographic and injection site. In this perspective article, we briefly review current microneedle designs, microfabrication methods, and industrialization strategies. We also provide an outlook where microneedles may become personalized according to a patient’s demographic in order to increase drug delivery efficiency and reduce healing times for patient-centric care.

## Introduction

The oldest and most common form of needling stems from tattooing, with the oldest recorded tools dating back over 3600 years [1] and the oldest recovered tattooed body being approximately 5000 years old [2]. However, it was not until the 1840s that Francis Rynd invented the first modern-day (i.e., synthetically fabricated) hypodermic needle [3]. Less than a decade later, hypodermic needles would be incorporated with syringe plungers to create a transdermal drug delivery device [4].

Clinicians rapidly adopted transdermal drug delivery (TDD) devices; however, this technique has drawbacks. The most known drawback to TDD needles is trypanophobia, a fear of needles. Roughly 3.5–20.0% of the general population suffers from trypanophobia to various degrees [5–6]. Additionally, healthcare workers are continuously at risk for sharps-related injuries, and hollow-bore needles account for 56% of all sharps injuries [7]. An estimated two million hospital-based workers suffer from work-related needle injuries, adding burdensome financial cost and infection risks to healthcare systems [8–9].

With the advent of advanced additive manufacturing techniques, we can miniaturize needles (microneedles) to overcome challenges with trypanophobia and hospital-based needle injuries. Today’s needles penetrate the deepest parts of the dermis, where discomfort or pain may occur [10]; however, today, we know that the stratum corneum is the only dermal layer clinicians need to penetrate to deliver non-intravenous medicine effectively [5,10–12]. To penetrate the stratum corneum, the length of a needle only needs to be of the order of tens to hundreds of micrometers, which gives rise to their name, microneedles.

## Perspective

**Figure 1:**
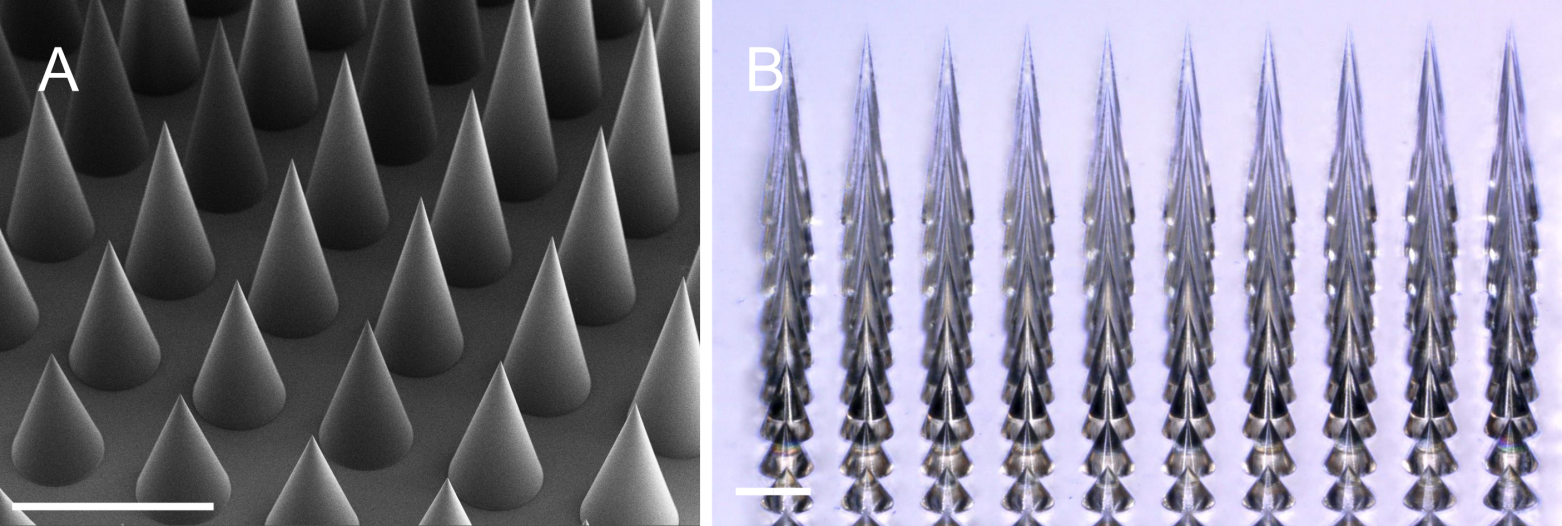
Conical microneedles with varying heights were printed via two-photon polymerization on a “Quantum X shape” lithography system, and the 10 × 10 microneedle array was printed in 130 min. The scale bar is 1 mm in both images. (A) Scanning electron microscopy image of microcones. (B) Optical microscopy image of transparent microcones.

According to current literature, TDD microneedles have relatively simple shapes, packing orders, and similar aspect ratios. A typical microneedle is either conical or pyramidal with a base of 100–300 μm, heights ranging from 600 to 1000 μm, and a base-to-base spacing between 100 and 500 μm (Figure 1). These simple geometries are advantageous for commercialization because the molds can be micromachined or etched and then used for mass production. One of the most common examples of commercialized TDD microneedles are over-the-counter anti-aging eye patches. Anti-aging eye patches consisting of 200–2000 dissolvable conical microneedles. These dissolvable microneedles are typically a variant of crosslinked hydrogels infused with hyaluronic acid, salicylic acid, caffeine, various vitamins (B3, C, and E), and a blend of various peptides.

Beyond cosmetic applications, microneedle patches are also being investigated for vaccine delivery. The most notable example of vaccine-loaded microneedles comes from the Australian company Vaxxas. Pty. Ltd. Vaxxas has developed a non-dissolving microneedle patch, called the Nanopatch [13–15]. In the Nanopatch, the microneedles are coated in a dry vaccine powder, and upon insertion, the microneedles leave pores in the skin where the powder particles can be bioabsorbed [14–15].

A closer inspection of TDD microneedles for cosmetic and medical applications reveals a lack of diversity when assessing their effectiveness. Namely, studies consist of small populations with participants of similar sex, age, body mass index, and ethnic background. For TDD via microneedles, it is crucial to consider that structural skin properties (e.g., transepidermal water loss, skin elasticity, dermal layer thicknesses, and ceramide content) differ among these groups [16–18], or else results give an incomplete picture. Several studies have also reported that the various demographic groups (e.g., ethnicity and age) heal at different rates [16,19]. Therefore, it is also critical to consider differences in skin penetration, drug absorption, and healing processes among different populations when assessing the effectiveness of drug-delivering microneedles.

In today's age of personalized medicine, it is possible to develop optimized microneedles for different populations and injection areas at scale. For example, microneedles are a promising alternative to oral and systemic medications for pain relief [20–22]. Chronic and acute pain can occur anywhere in the body; however, if we divide the body into mobile areas (i.e., joints) and passive areas (e.g., volar forearm), then a one-size-fits-all microneedle design may not satisfy the requirements for both applications [18]. This builds upon previous work from Rougier et al. [23], who demonstrated that drug absorption differs across the body. Their results agree with previous studies demonstrating that microneedles yield different penetrations depending on the injection site [24–25], whereby the closing of residual micropores and the pharmacokinetics may differ [26]. In the context of drug-loaded microneedle patches for joint-pain management, the microneedle patches will experience dynamic loads and may dislodge before delivering drugs. Thus, microneedles need to be engineered to bear the dynamic loads to last for the duration of treatment.

**Figure 2:**
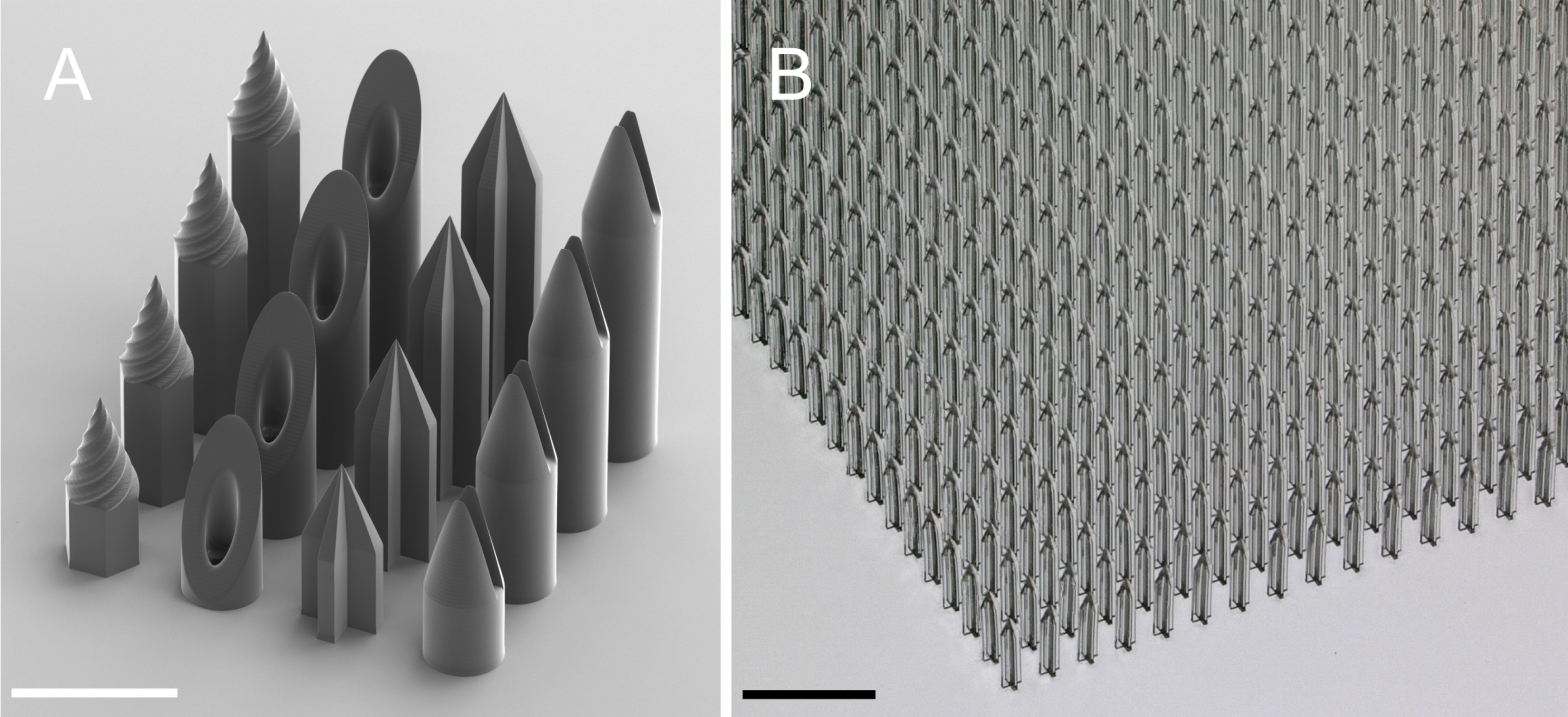
Various solid and hollow microneedle designs printed via two-photon polymerization on a “Quantum X shape” lithography system. (A) Scanning electron microscopy image of a 4 × 4 array consisting of both solid and hollow microneedles. The designs were inspired by Mizuno et al. [32] and Cordeiro et al. [29]. The scale bar is 500 μm. (B) Optical microscopy image of a large 2 cm × 2 cm array with 1746 individual microneedles. The microneedles are 1200 μm tall, 250 μm wide at the base, and spaced 500 μm apart. The large microneedle array was printed in 18 h. The scale bar is 1 mm.

Recent advances in microfabrication readily enable complex microneedle designs that can overcome these challenges [27]. Specifically, light-based 3D printing techniques such as stereolithography (SLA), digital light processing (DLP), and two-photon polymerization (2PP) simplify the rapid prototyping workflow when compared to traditional micro- and nanofabrication methods [28–31] (Figure 2). Thus, the greatest challenge is not fabricating but engineering improved microneedle designs to withstand evolving environments.

Researchers do not need to reinvent the wheel when thinking about new microneedle designs; rather they can adapt what nature has perfected over millennia. For instance, inspiration can come from insects [33–34], especially the approximately 100 species that have developed a preference for human hosts [35]. For example, mosquitos have an approximately 2 mm long proboscis that diverges into six stylets and easily penetrates skin [36–37], and there are several subspecies of mosquitos, such as *Aedes aegypti*, that have evolved to specialize in human hosts [35]. An adapted design would be advantageous for painless transdermal delivery of macromolecules or biological sampling [38]. Adult ticks, as another example, live on their hosts for 7–10 days and can occupy mobile areas, such as the back of the knee, without being disturbed. The strong attachment is possible because the backward-facing teeth lining their proboscis make it extremely difficult to remove, even under dynamic loads. A recent study from Liu et al. demonstrated improved tissue anchoring in barbed microneedles when compared to smooth microneedles [34]. Thus, it would be exciting to design microneedles, particularly those for pain management in joints, that are able to withstand a patient’s movements.

**Figure 3:**
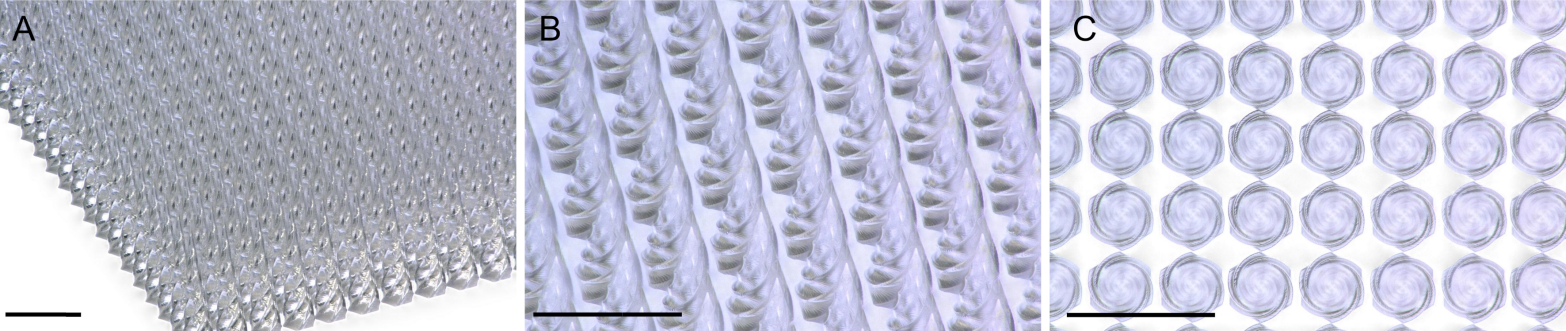
Simple twisted microneedles inspired by snake fangs and Bae et al. [39]. The microneedle array was printed via two-photon polymerization on a “Quantum X shape” lithography system. All scale bars are 500 μm. (A) Zoomed-out image of the microneedle array. (B) Closer inspection of optically transparent twisted microneedles. (c) Top-down view of the twisted microneedle array.

Bio-inspired designs do not need to be an exact replica of their animal muse to realize greater efficacy while also maintaining scalability. For example, snake fangs are hollow and asymmetrically grooved teeth optimized to deliver liquid venom. Researchers can use the bio-inspired hollow design to transdermally deliver drugs that must remain in liquid form during administration [39–40] (Figure 3). As with most bio-inspired microneedles, they require miniaturization, shape accuracy, and reproducibility for clinical applications.

**Figure 4:**
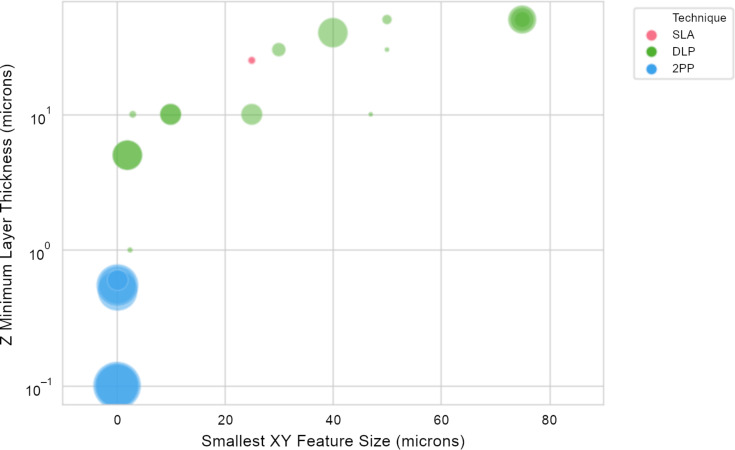
A graphical plot comparing the light-based printing microfabrication techniques SLA, DLP, and 2PP. This relationship plot compares the smallest feature sizes in the *XY* plane and the *Z* axis of commercial instruments. The bubble size represents the approximate cost of the instrument. We see a trend that when small feature sizes of the order of micrometers need to be obtained, the cost of the instrument substantially increases.

As mentioned above, light-based 3D printing (SLA, DLP, and 2PP) are the newest methods for fabricating microneedles. Each method has its own advantages and disadvantages; for brevity, we encourage readers to access previous review papers that cover in depth light-based fabrication techniques [41–43]. SLA and DLP are by far the most common techniques for fabricating microneedles, with approximately eight times as many publications as publications regarding microneedles fabricated via 2PP. We hypothesize that the availability SLA and DLP sytems, the low cost, and the sheer number of systems present at any given institution, are the main driving factors behind the difference in publication quantity. SLA or DLP 3D printers may only cost a few thousand dollars (2500+ USD) when new, but these cost-effective printers are aimed at hobbyists and lack the resolution necessary for microneedle development (Figure 4). Professional SLA or DLP printers are more suitable for microneedle development because they can achieve feature sizes of the order of a few micrometers; however, these professional systems can cost upwards of 250,000+ USD.

The financial differences for acquiring a commercial 2PP system are not so different from those regarding professional-grade DLP or SLA printers. On average, a commercial 2PP instrument may cost a few hundred thousand dollars (400,000+ USD) for a single device (Figure 4). Custom-built 2PP systems are also an option; these are custom systems either retrofitted onto an existing microscope [44] or developed on optical tables [45–46] in the lab to keep the cost low. Commercial retrofits (e.g., positioning stages or electronics) can add up to 50,000+ USD, and this price does not include the femtosecond laser or anti-vibration systems. Custom-built 2PP systems also require extensive optical expertise for the initial installation and are typically a major milestone for several doctoral students. In general, custom-built 2PP systems are constantly evolving instruments to enable one or two particular applications at a time before they are modified again for one or two different applications [46]. In this context, custom-built 2PP systems, while potentially cheaper than commercial instruments, are not particularly suitable for microneedle development and clinical translation.

Despite SLA and DLP being cost-effective entry points for fabricating bio-inspired microneedles, both techniques often suffer from rough surfaces and poor shape accuracy. Specifically, SLA and DLP 3D printers suffer from the staircase effect, which is an artifact from slicing the computer-aided design (CAD) into layers [47–48]. The staircase effect leads to increased surface roughness of the order of micrometers, and high surface roughness on the microneedles will require substantially more pressure to penetrate the skin. These slicing artifacts ultimately impede quality control [49]. Finally, DLP- and SLA-fabricated microneedles are often dull with a low aspect ratio [50], and this again affects their performance (e.g., penetration).

Hence, 3D printing via 2PP is currently the best commercially available microfabrication method to enable miniaturization, shape accuracy, smooth surfaces, and high aspect ratios [51–54]. In the last decade, 2PP instruments have evolved from slow (albeit versatile) microfabrication instruments (e.g., the first generation 2008–2012 Photonic Professional) suitable for proofs of concept to industrialized 3D printers (e.g., Nanoscribe Quantum X platforms) producing polymer masters. The increased scan speed and throughput are the result of new supporting technologies coming to market, such as replacing piezoelectric stages with galvanometric mirrors. These new technologies continue to decrease the gap in volumetric throughput between DLP or SLA printers and 2PP instruments.

In conjunction with new hardware, there have also been incremental changes in 2PP fabrication strategies. These strategies are based on voxel (volumetric pixel in *x*,*y*,*z*) control. Current 2PP software can be utilized to classify parts of the structure for either fine or coarse slicing, such as Nanoscribe’s “Smart Slicing” and “Shell & Scaffold” [55]. “Smart Slicing” can be applied to optimize print times for microneedle arrays by defining bulks areas, such as the base, with coarse slicing, and assigning finer slicing to the penetrating tips. “Shell & Scaffold” is leveraged to print hollow rather than solid structures, thereby polymerizing less internal volumes to speed up the printing process. Important to note, structures made by “Shell & Scaffold” require a post-development UV curing for a few minutes to polymerize the internal photoresin.

A third fabrication strategy leverages the combination of DLP and 2PP fabrication techniques. Sarker et al. [56] recently demonstrated the strength of combining fabrication techniques for microneedles. They used DLP to fabricate the bulky base and 2PP for microneedle fabrication, akin to Nanoscribe’s “Smart Slicing” feature. All strategies, either software or combined fabrication methods, drastically reduce printing time and, ultimately, production cost.

Even with software and hardware advances, 2PP is still a layer-by-layer approach, similar to DLP and SLA, such that we see the staircase effect, albeit on a smaller scale. The staircase effect affects the surface quality and shape accuracy for complex geometries found in bio-inspired microneedles. It may also limit design freedom, such as the structural quality of overhanging features. Taking inspiration from similar challenges in microoptic fabrication, grayscale lithography offers a solution to mitigate the staircase effect. Grayscale lithography is a novel approach in photolithography for 2.5D patterning (*x*,*y*,*z*) with ultrasmooth surfaces that exhibits improved shape accuracy [57–58]. In 2019, Nanoscribe GmbH & Co launched the Quantum X platform that commercialized maskless grayscale lithography for microoptics made via 2PP, a process called “Two-Photon Grayscale Lithography” (2GL^®^) [59–60]. 2GL^®^ differs from traditional 2PP and 1PP lithography because the laser or exposure dosage in individual voxels is controlled in three dimensions with high spatial resolution, which lends itself to continuous rather than discrete printing [59,61]. A key result of 2PP grayscale printing is that the technique is, on average, five to ten times faster than the common 2PP layer-by-layer approach [52]. Thiel et al. successfully applied 2GL^®^ to free-standing 3D structures for the first time [62]. They also demonstrated that 3D printing by 2GL^®^ was faster with better surface quality and shape accuracy than 2PP layer-by-layer methods [62]. Currently, Nanoscribe GmbH & Co is exploring 2GL^®^ in three dimensions, and we look forward to the technique being commercially available to researchers and industry.

While microfabrication techniques have improved over the years in terms of cost, throughput, and quality, there is still a “valley of death” to cross when translating microneedle technologies from the lab to manufacturers. Specifically, proofs of concept require only a few pieces to be printed, compared to the millions or billions needed to be produced for a commercial product. For example, more than three billion people have received at least one COVID-19 vaccination [63]. Harro Höfliger and Vaxxas estimated that they would have had to produce tens of millions of Nanopatches per week for COVID-19 vaccination [64–65]. If companies were to personalize and optimize microneedles (including design, dosage, and vaccine composition) for COVID-19 or influenza vaccination based on ethnicities [66], those manufacturers would have to produce millions of pieces for each ethnic population.

Today, there are mass production methods using light-based 3D printing that mitigate the risks associated with scaling up production, namely (1) polymer masters for solid microneedle replication and (2) direct printing of hollow microneedles

The first approach to fabricate solid microneedles from polymer masters is more favorable for mass production because soft and hard molds can be generated rapidly [50,67]. From these molds, medical device manufacturers can mass-produce drug-loaded microneedle arrays, which lowers the overall production cost [50]. The original polymer masters can be reused to generate new molds as necessary for production. Important to note, there are caveats regarding the lifetime of the polymer masters that depend on the exact replication technique, such as soft PDMS molding [68] or plastic microinjection molding [10]. The lifetime of a polymer master needs to be considered for the overall cost of production and product. Using polymer masters and mold techniques also follows accepted and well-established processes from regulatory bodies and manufacturers in adjacent medical market segments. This method also enables the production of either dissolving or non-dissolving drug-loaded microneedles.

The second approach is better suited for pure medical devices, that is, microneedles that are not loaded with drugs. While this article has not discussed the applications of hollow microneedles, it is worth mentioning that hollow microneedles are most often directly printed onto the medical device and aligned to specific features, such as pores, on the device. Direct microneedle printing has its own set of extensive requirements (e.g., biocompatibility of the material, mechanical robustness, and surface adhesion). Also, FDA’s 510(k) criteria need to be considered early on in the conceptual phase. Furthermore, direct fabrication is more costly than creating molds from a polymer master, and the fabrication cost is a crucial factor when a new medical device is launched onto the market.

## Conclusion

Microneedles are emerging as a new medical device for administering drugs and collecting biological fluids, all while reducing sharps-related risks to healthcare professionals. To date, microneedle patches are relatively simple with a one-size-fits-all approach, regardless of patient demographic and injection site. 3D microfabrication instruments enable the investigation of complex microneedle shapes and arrangements that can be personalized for patients. Microfabrication instruments based on 2PP can rapidly prototype bio-inspired microneedles and can be utilized in production. Importantly, using the same instrument for prototyping and production is advantageous for reducing design transfer and going to market faster. Microneedle fabrication is a rapidly evolving field that is overcoming traditional fabrication challenges and opening the door for personalized medicine.

## Methods

All microneedle arrays in the presented images were 3D printed on a “Quantum X shape” lithography system (Nanoscribe GmbH & Co, Karlsruhe, Germany) following printing and post-printing protocols from NanoGuide. Post-printing protocols begin by removing unpolymerized material via two-step washing with either propylene glycol methyl ether acetate or mr-Dev for 15–20 min, followed by an isopropyl alcohol bath for 2–5 min. Afterwards, the microneedles were allowed to dry in air before UV curing for 20–40 min.

The microneedles were fabricated from proprietary and commercially available negative-tone IPX-Q and negative-tone IP-S with the medium (ZEISS 25x NA 0.8 objective) and large (ZEISS 10x NA 0.8 objective) feature sets. IPX-Q and IP-S are methacrylate photoresins; after polymerization, both materials are non-cytotoxic, as certified via external ISO-10993-5 commissioning.

IP-S is a photoresin optimized for printing smooth microoptics with the medium solution set (ZEISS 25x NA 0.8 objective). However, IP-S has been extensively used for microneedle molds and direct microneedle fabrication. IP-S is compatible with both Photonic Professional and Quantum X systems. IPX-Q is the newest formulation from the original IP-Q. It is optimized for printing 3D structures with the large solution set (ZEISS 10x NA 0.8 objective) on the Quantum X systems. IPX-Q is preferred over IP-S for microneedle applications as IPX-Q, on average, prints faster than IP-S. This is a result of the IPX-Q formulation being optimized for 3D structures.

Nanoscribe GmbH & Co is not a manufacturer of either medical devices or molds made from polymer masters. Users of the Nanoscribe Photonic Professional systems have extensively used IP-S and IP-Q for direct microneedle fabrication and molding. Given IPX-Q is the newest formulation of IP-Q, we expect similar results to those previously published.

We utilized Google Scholar to calculate the number of microneedle publications from 2015 to 2023 across different techniques. We did not include citations and patents in this search. For DLP and SLA microfabrication we used the keywords “microneedle DLP”, “microneedle Digital Light Processing”, “microneedle SLA”, and “microneedle Stereolithography”. Similarly, we used the following keywords for 2PP-fabricated microneedles: “microneedle 2-photon polymerization”, “microneedle 2-photon polymerisation”, “microneedle 2PP”, “microneedle TPP”, and “microneedle multiphoton lithography”.
